# Timely sown maize hybrids improve the post-anthesis dry matter accumulation, nutrient acquisition and crop productivity

**DOI:** 10.1038/s41598-023-28224-9

**Published:** 2023-01-30

**Authors:** R. R. Zhiipao, Vijay Pooniya, Niraj Biswakarma, Dinesh Kumar, Y. S. Shivay, Anchal Dass, Ganapati Mukri, K. K. Lakhena, R. K. Pandey, Arti Bhatia, Prabhu Govindasamy, Anamika Burman, Subhash Babu, R. D. Jat, A. K. Dhaka, Karivaradharajan Swarnalakshmi

**Affiliations:** 1 Agronomy, ICAR-Indian Agricultural Research Institute (IARI), New Delhi, 110 012 India; 2 Genetics, ICAR-Indian Agricultural Research Institute (IARI), New Delhi, 110 012 India; 3 Plant Physiology, ICAR-Indian Agricultural Research Institute (IARI), New Delhi, 110 012 India; 4 Environment Science, ICAR-Indian Agricultural Research Institute (IARI), New Delhi, 110 012 India; 5 Chaudhary Charan Singh Haryana Agricultural University (CCSHAU), Hisar, Haryana 125004 India; 6 Microbiology, ICAR-Indian Agricultural Research Institute (IARI), New Delhi, 110 012 India

**Keywords:** Agroecology, Climate-change ecology, Ecosystem ecology, Climate sciences

## Abstract

Delayed sowing of maize hybrids could exacerbate the capability of maximizing the yield potential through poor crop stand, root proliferation, nutrient uptake, and dry matter accumulation coupled with the inadequate partitioning of the assimilates. This study appraised the performance of five recent maize hybrids viz., PMH-1, PJHM-1, AH-4158, AH-4271, and AH-8181 under timely and late sown conditions of the irrigated semi-arid ecologies. Timely sowing had the grain and stover yields advantage of 16–19% and 12–25%, respectively over the late sown maize hybrids. The advanced hybrids AH-4271 and AH-4158 had higher grain yields than the others. During the post-anthesis period, a greater dry matter accumulation and contribution to the grain yield to the tune of 16% and 10.2%, respectively, was observed under timely sown conditions. Furthermore, the nutrient acquisition and use efficiencies also improved under the timely sown. The nutrient and dry matter remobilization varied among the hybrids with AH-4271 and PMH-1 registering greater values. The grain yield stability index (0.85) was highest with AH-4158 apart from the least yield reduction (15.2%) and stress susceptibility index (0.81), while the maximum geometric mean productivity was recorded with the AH-4271 (5.46 Mg ha^−1^). The hybrids AH-4271 and PJHM-1 exhibited improved root morphological traits, such as root length, biomass, root length density, root volume at the V5 stage (20 days after sowing, DAS) and 50% flowering (53 DAS). It is thus evident that the timely sowing and appropriate hybrids based on stress tolerance indices resulted in greater yields and better utilization of resources.

## Introduction

Maize (*Zea mays* L.), the third most cultivated crop after rice and wheat, can be grown in various soils and climates due to its versatility^1,2^. However, delayed sowing after an optimum time can result in reduced yields anomalies, due to aberrant weather events and irregular rainfall. Timely sowing is crucial for maximizing the yield of maize, and growers are concerned about the yield response of maize to sowing dates^3^. In addition, sowing at an optimum time could enhance the profitability of maize by improving the yields, as the crop has extended period to photosynthesize as well as avoid the artificial grain drying at the end of the crop cycle due to various environmental stresses^4^. The increased maize production is the result of improved agronomic management, varietal development^5^, and advances in plant protection measures. Furthermore, yield increment in a particular area is governed by the timely sowing, due to differences in climate and length of the growing season^6^. The supply of assimilate to grain in cereals derived from current assimilation, which supplies directly to kernels, and then remobilises the temporarily stored assimilates in the vegetative plant parts^7,8^. Reserve assimilates storage by a stem is strongly influenced by the growing conditions from emergence to the anthesis. Therefore, the grain yield could be buffered by the reserves accumulated in the vegetative portions of the plant pre-anthesis against the unfavourable current assimilation, particularly during the grain filling^8^.

In rice, an extended period of grain filling is the main determinant of grain yield, as it leads to a higher accumulation of dry matter because of greater cumulative mean temperatures and greater solar radiation interception^9,10^. The translocation of dry matter stored in the vegetative plant parts pre-anthesis and accumulation of photosynthates post-anthesis determined the grain yield of rice^11^. The dry matter translocated to grains during grain filling from the accumulated dry matter pre-anthesis and accumulation between flowering to physiological maturity acts as a function of grain yield in maize^12,13^, and differs among crop species and nutrient inputs^14^. In addition, it has been reported that ~ 85% of total grain dry weight was derived from the photo-assimilation during the grain filling period in maize^11^. Thus, the timely sowing of the genotypes improved the accumulation of photo-assimilates and their remobilization post-anthesis to grains. Further, timely sowing of maize hybrids could results in better interception of the photosynthetically active radiation leading to improved growth and development of the crop.

The identification of genotypes tolerant to stress and non-stress environment have been reported in crops using the indices, such as, stress susceptibility index (SSI), tolerance index (TOL), yield stability index (YSI), and geometric mean productivity (GMP)^15,16^. The SSI distinguishes the genotypes showing a minimum reduction in yield under the stress against the non-stress condition^17^, but it fails to identify the genotypes with high yield and stress-tolerant^16^. Further, the TOL indicates that the higher the TOL value more is tolerant to stress resulting in a higher yield potential of the genotypes^18^. Similarly, YSI is yet another index for identifying the stability of genotypes based on the yield under the stress and non-stress environment^19^. The GMP is similar to STI, wherein it indicates that the genotypes with higher GMP could be selected for both the stress and non-stress environment^15^. The above indices for the selection of genotypes have been reported mostly for wheat and other cereals by inducing an environment susceptible to the stress. Therefore, identifying the maize genotypes with higher yield potential under both stress and non-stress (timely and delayed sowing) environments would be a more robust and efficient approach.

In modern high-yielding maize hybrids, nitrogen use efficiency (NUE) has a negative correlation with the grain N concentration (GNC), defined as the grain yield per unit of nitrogen present in soil and applied fertilizer^16,20,21^. The decline in GNC could be attributed to the leaves staying green, which depends on the enhanced N absorption from the soil post-anthesis and less remobilization from the vegetative plant parts^22,23^. While genotypes with greater NUE under varied conditions can be beneficial in terms of protecting the environment, this should be considered when developing new varieties / hybrids and when recommending fertilizers such as N^24^. Moreover, greater N accumulation post-anthesis has a positive correlation with grain yield in rice, indicating that the post-anthesis N-accumulation plays an essential role in expanding the grain yield^25^. Subsequently, adequate concentration of phosphorus (P) is imperative for maintaining a high photosynthesis rate and enhancing the dry matter accumulation^26^. In addition, the extended period of leaf photosynthesis enhanced the grain yield but the trade-off with N and P remobilization in leaves during the grain filling as it shortens leaf photosynthesis^27^. In contrast, potassium (K) differs from P and N as it functions in various enzyme activation, synthesis of protein, maintaining osmotic balance and soluble metabolites translocation within plant tissue^28^. The hidden half of the plant (root systems) greatly influenced the performance of the above-ground portion particularly the formation of grain. Thus, the genotype with better rooting pattern could enhance the acquisition of limited resources (water, nutrients, etc.) and improved the yielding potential of the crop. Further, the root system architecture differs among genotypes, and so too does the nutrients and water uptake and ultimately the yield. Moreover, genotypes with improved root growth parameters and proliferation proportionately partitioned the captured resources and enhanced the yields^29,30^.

High-yielding hybrids are a boon for growers, but they require higher inputs as well as timely monitoring of all management practices. Hence, it is necessary to evaluate the performance of advanced/recent hybrids under the optimum nutrient management with varying sowing dates to know the productivity potential. This field study investigates the productivity, translocation and accumulation of dry matter besides the nutrient use-efficiencies and stress tolerance indices under the timely and late sowing of maize hybrids, in irrigated semi-arid ecologies.

## Results

### Harvest index, grain and stover yields

On an average, the timely sown genotypes recorded 5–10% higher number of cobs per ha over the delayed sowing. While, among the hybrids, advanced hybrid AH-4271 recorded significantly a greater number of cobs ha^−1^. Harvest index (HI) did not differ significantly under different sowing times, but advanced hybrids AH-4158 and AH-4271 showed significantly higher HI. Timely sowing gave a significantly stover yield advantage of 12–25.9% over the late sowing. PMH-1 and PJHM-1 had 5.2–16.9% and 5–11% greater stover yields than other hybrids in 2020 and 2021, respectively. With timely sowing of hybrid maize, grain yield increased by 16.7–19.2% compared to late sown hybrids. Hybrid AH-4271 had the highest grain yield, being similar to AH 4158, but significantly greater than the other hybrids (Table 1).

**Table 1:** Number of cobs, harvest index and yields of maize genotypes under timely and late sown conditions.

Treatment	Cobs ha^-1^		HI		Stover yield (Mg ha^-1^)		Grain yield (Mg ha^-1^)	
	2020	2021	2020	2021	2020	2021	2020	2021
Sowing time (S)								
Timely	64625a	66625a	0.41a	0.39a	7.4a ± 0.70	8.5a ± 0.77	5.2a ± 0.63	5.4a ± 0.91
Late	61375b	59419b	0.39a	0.41a	6.5b ± 0.74	6.3b ± 0.47	4.2b ± 0.43	4.5b ± 0.77
Genotypes (G)								
PMH1	56562d	56093d	0.38c	0.36b	7.7a ± 1.04	7.2c ± 1.63	4.8b ± 0.62	3.9c ± 0.54
PJHM 1	64375bc	65000b	0.39bc	0.37b	6.4d ± 0.29	8.0a ± 1.36	4.1c ± 0.68	4.7b ± 0.40
AH 4158	64687b	66718a	0.43a	0.45a	6.7 cd ± 0.43	7.0c ± 0.98	5.0ab ± 0.55	5.7a ± 0.60
AH 4271	66250a	66985a	0.42ab	0.44a	7.3b ± 1.00	7.1c ± 0.51	5.3a ± 0.84	5.8a ± 0.92
AH 8181	63125c	60312c	0.39c	0.37b	6.9c ± 0.62	7.6b ± 1.80	4.3c ± 0.47	4.5bc ± 0.65
S × G	**	**	ns	**	*	**	*	**

Within sowing time and genotypes, different letters indicate the significant difference (*p* ≤ *0.05*); HI: Harvest index, The S × G implies the interaction between sowing time and genotypes; **Significant at *p* ≤ *0.01*; *Significant at *P* ≤ *0.05*; ns: not significant.

### Yield attributes

The grains cob^−1^ under the timely sown was higher in 2020, but similar to the late sown in 2021. The highest grains cob^−1^ was recorded with AH-4271. Further, heavier cobs were harvested in timely sown than in late sown crop, though being similar in 2021. Among hybrids, the PMH-1 recorded the maximum cob weight, being similar to the AH-4158 and AH-4271. The timely sown crop produced 22.7% and 8.2% higher grain weight cob^−1^ over the late sown crop during 2020 and 2021, respectively. While, the grain weight among the hybrids were comparable, except AH-8181 in 2020, but in 2021 PMH-1 recorded the highest weight over other hybrids (Table 2).

**Table 2:** Yield attributes of maize genotypes under timely and late sown conditions.

Treatment	Grains cob^−1^		Cob weight (g cob^−1^)		Grain weight cob^−1^ (g)	
	2020	2021	2020	2021	2020	2021
Sowing time (S)						
Timely	478.2a	476.1a	159.7a	153.0a	125.8a	123.7a
Late	432.8b	475.7a	119.1b	149.7a	97.2b	113.5b
Genotypes (G)						
PMH 1	451.5b	507.9ab	155.1a	158.5a	118.6a	124.8a
PJHM 1	403.4c	417.5c	127.5b	151.5ab	104.0ab	116.9bc
AH 4158	509.6a	496.7b	144.4ab	149.2ab	114.8ab	119.4b
AH 4271	527.9a	557.4a	140.5ab	144.8b	118.0ab	112.9c
AH 8181	385.2c	399.6c	129.4b	152.5ab	102.2b	119.1b
S × G	ns	ns	ns	ns	ns	ns

Within sowing time and genotypes, different letters indicate the significant difference (*p* ≤ *0.05*); HI- Harvest index; The S × G implies the interaction between sowing time and genotypes; **Significant at *p* ≤ *0.01*; *Significant at *p* ≤ *0.05*; ns- not significant.

### Accumulated dry matter, its translocation and contribution to the grain yields

**Figure 1:**
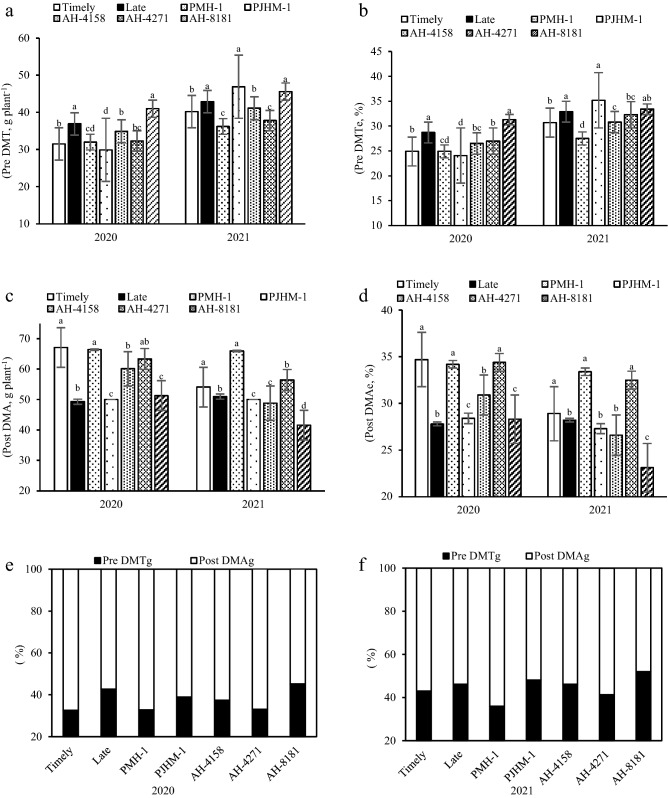
Pre DMT- pre-anthesis dry matter translocation (**a**), Pre DMTe- pre-anthesis dry matter translocation efficiency (**b**); Post DMA- post-anthesis dry matter accumulation (**c**), Post DMAe- post-anthesis dry matter accumulated efficiency (**d**); Pre DMTg- pre-anthesis dry matter translocation and Post DMAg- post- anthesis dry matter accumulation contribution to grain yield (**e**, **f**). Within years, sowing time, and genotypes, different letters on the individual bars of a figure indicate significant difference (*p* ≤ 0.05).

In 2020 and 2021, the late sowing led to a 6.3–14.6% increase in the dry matter translocation before anthesis (Fig. 1a). Pre DMT translocation was the greatest with AH-8181 and PJHM-1. The effectiveness of dry matter translocation (Pre DMTe) was also higher under late sown than the timely sown crop (Fig. 1b). While AH-8181 had the greatest Pre DMTe in 2020, PJHM-1 and AH-8181 in 2021. On the other hand, hybrids sown at the right time accumulated 26.5% (2020) and 5.7% (2021) greater dry matter after anthesis (Post DMA) (Fig. 1c). PMH-1 and AH-4271 recorded the highest Post DMAs. Post anthesis dry matter accumulation efficiency (Post DMAe) under timely sowing was 2.4–19.9% greater than the late sowing (Fig. 1d). PMH-1 and AH-4271 had a greater Post DMAe than the other hybrids. In 2020, the contribution of pre-anthesis dry matter translocation to grain yield (Pre DMTg) was higher for the late sowing (Fig. 1e), while in 2021, it was the same for both the sowing dates (Fig. 1f). In both the years, AH-8181 gave maximum Pre DMTg. Post-anthesis dry matter accumulation contributed 5.6–14.7% more to grain yield under the timely sowing compared to the late sowing. Hybrids AH-4271 and PMH-1 had similar Post DMAg in 2020, while PMH-1 had the maximum in 2021.

### Nutrient concentration in different plant parts at flowering and maturity

Under the timely sown conditions, leaf and stem –N concentration at flowering was 2.9% and 8.8% greater than in the late sown, while hybrids didn’t differ significantly for leaf -N (Table 3). However, AH-4148 and AH-4271 had greater stem –N. Contrary to the N, the late sown crop had greater leaf and stem -P than timely sown, whereas hybrids had similar stem –P. For leaf –P concentration, PMH-1 being similar to AH-4158 and AH-8181, but greater than PJHM-1 and AH-4271. Leaf -K concentration in timely sown maize was 15.8% greater than that in the late sown. AH-4271 had greater leaf -K than that of PMH-1 and AH-8181, being similar to PJHM-1 and AH-4158. At maturity, the leaf and stem –N under timely sown was 10.7% and 3.8% greater, respectively over the late sown. The late seeded crop had higher leaf –P concentration than the timely sown, and the hybrid AH-8181 accumulated greater leaf –P than the other hybrids. Similarly, the leaf –P concentration was the highest in AH-8181, being comparable to PMH-1 and PJHM-1. While grain P in the hybrids was similar apart from the AH-4158. When sown early, the K concentration (leaf, stem, and grain) in maize hybrids was greater than when sown late. PJHM-1 had the highest leaf and grain –K concentration, while AH-8181 greater stem –K concentration.

**Table 3:** Nutrients concentration in different plant parts of maize genotypes at 50% flowering and at maturity under timely and late sown conditions.

Treatment	Nutrient concentration (%) at 50% flowering						Nutrient concentration (%) at maturity								
	Leaf N	Stem N	Leaf P	Stem P	Leaf K	Stem K	Leaf N	Stem N	Grain N	Leaf P	Stem P	Grain P	Leaf K	Stem K	Grain K
Sowing time (S)															
Timely	2.04a	0.80a	0.10b	0.08b	0.76a	0.83a	1.40a	0.53a	1.33a	0.07a	0.04a	0.22a	0.66a	0.72a	0.31a
Late	1.98b	0.73b	0.13a	0.10a	0.64b	0.82a	1.25b	0.51b	1.36a	0.07a	0.05a	0.23a	0.58b	0.69b	0.26b
Genotypes (G)															
PMH 1	2.02a	0.78b	0.13a	0.09a	0.67c	0.82a	1.0a	0.50a	1.30a	0.07ab	0.04b	0.23ab	0.60b	0.69bc	0.30b
PJHM 1	1.93a	0.75c	0.09b	0.10a	0.72ab	0.85a	1.23b	0.54a	1.37a	0.07abc	0.04b	0.25a	0.64a	0.67c	0.32a
AH 4158	2.06a	0.83a	0.12a	0.08a	0.71abc	0.80a	1.41a	0.50a	1.39a	0.06bc	0.04c	0.20b	0.62ab	0.71b	0.28c
AH 4271	2.03a	0.80a	0.10b	0.08a	0.72a	0.80a	1.41a	0.55a	1.32a	0.06c	0.03d	0.23ab	0.62ab	0.70b	0.28c
AH 8181	2.00a	0.66d	0.12a	0.09a	0.68bc	0.85a	1.19b	0.52a	1.35a	0.08a	0.05a	0.22ab	0.59b	0.75a	0.26c
S × G	ns	**	**	*	**	**	**	*	ns	**	**	**	**	**	**

N: Nitrogen, P: Phosphorus, K: Potassium. The nutrient concentration given in the table is the mean of 2 years (i.e., 2020 and 2021). Within sowing time and genotypes, different letters indicate the significant difference (*P* ≤ 0.05); the S × G implies the interaction between sowing time and genotypes; **Significant at *p* ≤ 0.01; *Significant at *p* ≤ 0.05; ns: not significant.

### Nitrogen translocation and uptake

**Figure 2:**
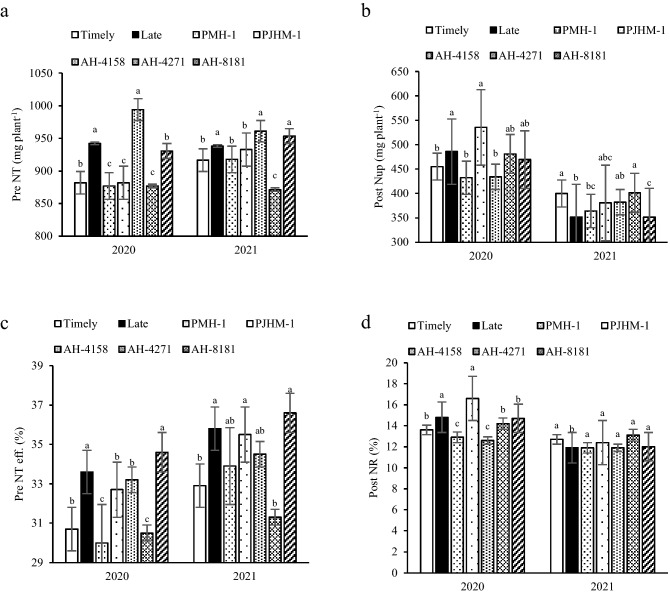
Pre NT- pre-anthesis Nitrogen translocation (**a**), Post NuP- post-anthesis Nitrogen uptake (**b**), Pre NT-eff.- pre-anthesis Nitrogen translocation efficiency (**c**), Post NR- ratio of post-anthesis N uptake to total N accumulation (**d**). Within years, sowing time, and genotypes, different letters on the individual bars of a figure indicate significant difference (*p* ≤ 0.05).

Pre-anthesis N translocation in late-sown crop was 2.3–6.4% lower than in the timely-sown crop (Fig. 2a). In both the years, hybrid AH-4158 exhibited significantly a greater translocation than other hybrids and was comparable to the hybrid AH-8181. The translocation efficiency (Pre NT eff.) was higher with the late-sown to the tune of 8.1–8.6% (Fig. 2c). The hybrid AH-8181 achieved the best efficiency among the hybrids. After anthesis under the late sown condition, the N uptake (Post Nup) was 6.3% greater; however, timely sown had 12% greater uptake in 2021 (Fig. 2b). As for N uptake, PJHM-1 was comparable to the AH-4271 and AH-8181, but significantly different from the PMH-1 and AH-4158. In 2021, the uptake of N by AH-4271 was 9.3 and 12.4% greater than that of PMH-1 and AH-8181. The proportion of N uptake after anthesis to total N accumulation (Post NR) varied with years and sowing times (Fig. 2d). In 2020, PJHM-1 had significantly a greater Post NR than the other hybrids, but in 2021 the hybrids didn’t differ significantly.

### Phosphorus (P) translocation and uptake

**Figure 3:**
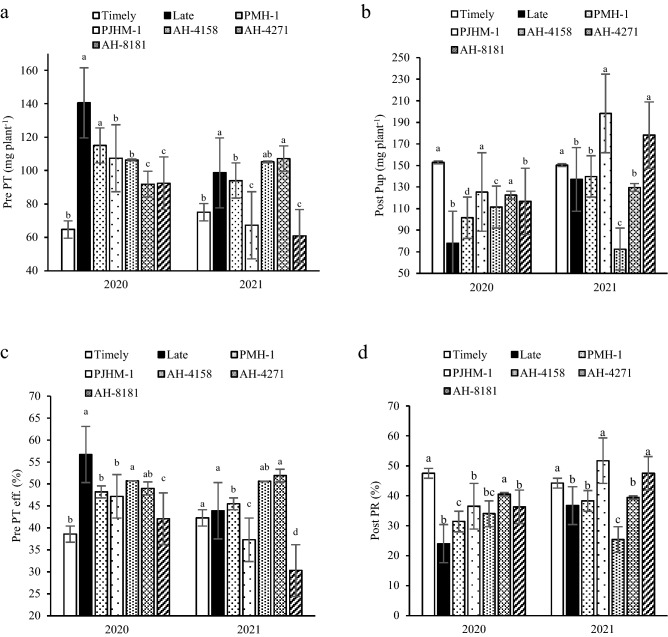
Pre PT- pre-anthesis phosphorus translocation (**a**), Post PuP- post-anthesis phosphorus uptake (**b**), Pre PT-eff.- pre-anthesis Phosphorus translocation efficiency (**c**), Post PR- ratio of post-anthesis P uptake to total P accumulation (**d**). Within years, sowing time, and genotypes, different letters on the individual bars of a figure indicate significant difference (*p* ≤ 0.05).

Pre-anthesis P translocation under the late sown crop was significantly greater than timely sown crop (Fig. 3a). PMH-1 and AH-4271 had significantly greater pre-anthesis translocation rates than the other hybrids. Under late sowing, the translocation efficiency (Pre PT efficiency) was greater than the timely sowing (Fig. 3b). In comparison with AH-4158, AH-4271 had significantly a higher Pre PT efficiency. Under timely sown conditions, the P uptake after anthesis (Post Pup) was 8.9–48.9% greater than the late sown crop (Fig. 3c). In 2020, PJHM-1 and AH-4271 were similar, but significantly greater than other hybrids, whereas in 2021, it was PMH-1 and AH-8181. Under timely sowing, total P accumulation (Post PR) increased by 16.9–49.5% compared to the late sowing (Fig. 3d). However, among the hybrids, in 2020 Post PR was highest with AH-4271, and in 2021, the PJHM-1 and AH-8181 were comparable, but significantly more than the other hybrids.

### Potassium (K) translocation and uptake

**Figure 4:**
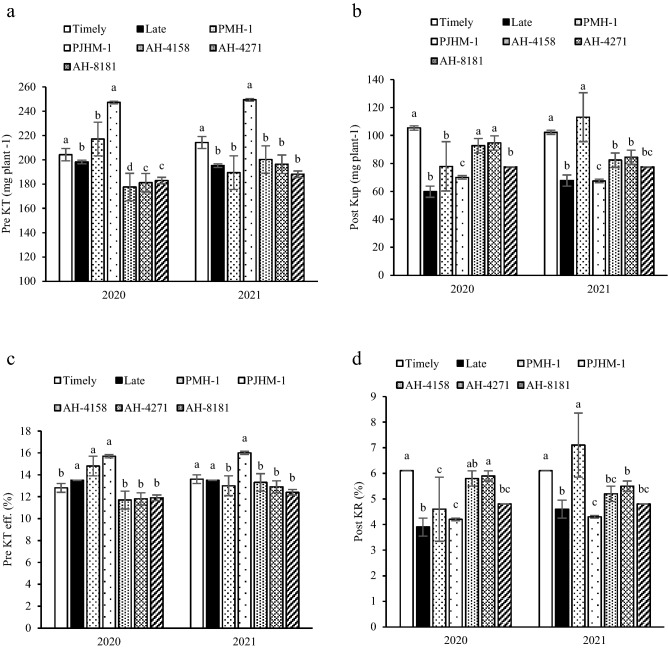
Pre KT- pre-anthesis potassium translocation (**a**), Post KuP- post-anthesis potassium uptake (**b**), Pre KT-eff.- pre-anthesis potassium translocation efficiency (**c**), Post KR- ratio of post-anthesis K uptake to total K accumulation (**d**). Within years, sowing time, and genotypes, different letters on the individual bars of a figure indicate significant difference (*p* ≤ 0.05).

The K translocation (Pre KT), its uptake after anthesis (Post Kup), and the ratio of K uptake to total K accumulation (Post KR) under timely sown were significantly greater than the late sown (Fig. 4a–d). Compared to the late sowing, timely sown had an increments of 2.9–8.9% (Pre KT), 33.7–43.2% (Post Kup), and 24.5–36% (Post KR), respectively. PJHM-1 had the highest Pre KT and Pre KT efficiency than the other hybrids. Post Kup for AH-4158, AH-4271 and PMH-1 had recorded greater values than other hybrids. In 2020, AH-4158 and AH-4271 had comparable Post KRs, but AH-4271 recorded significantly more than other hybrids, however in 2021, PMH-1 recorded a statistically higher Post KR.

### Contribution of nutrient translocation and uptake to the grain yields

**Figure 5:**
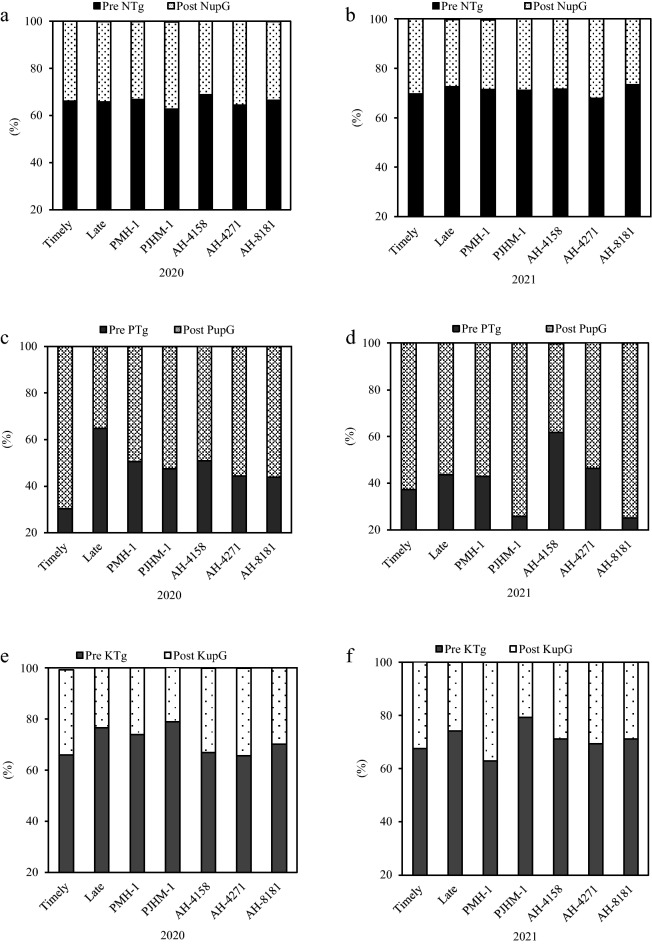
Pre NTg- Pre-anthesis nitrogen translocation and Post NupG- Post-anthesis nitrogen uptake contribution to grain yield (**a**, **b**); Pre PTg- Pre-anthesis phosphorus translocation and Post PupG- Post-anthesis phosphorus uptake contribution to grain yield (**c**, **d**); Pre KTg- Pre-anthesis potassium translocation and Post KupG- Post-anthesis potassium uptake contribution to grain yield (**e**, **f**).

Despite of varying sowing dates and years, N translocation before anthesis did not affect the grain yields. Both PMH-1 and AH-4158 showed greater contributions to the pre-anthesis N translocation than did AH-4271 (Fig. 5a,b). With late sown conditions, pre-anthesis P translocation was 14.8–53.3% greater than the timely sown crop (Fig. 5c,d). Pre-anthesis translocation was largely accounted by AH-4158, followed by PMH-1. A greater K translocation was recorded under the late-sown conditions (Fig. 5e,f). Meanwhile, PJHM-1 outperformed the other hybrids; timely sown hybrids contributed 10.8% more N to the grain yield from post-anthesis uptake (Fig. 5a). In 2021, AH-4271 increased the post-anthesis uptake of N by 9.7–16.3%. Also, timely sowing increased the P uptake by 49.4% and 10.3%, respectively in 2020 and 2021 (Fig. 5c,d). P uptake contributions were higher for AH-4271 and AH-8181 in 2020, whereas in 2021 they were PJHM-1 and AH-8181. Through post-anthesis K uptake, timely sowing contributed 21.1–29.7% more to grain yield over the late sowing (Fig. 5e,f). AH-4158, AH-4271 and PMH-1 contributed most to the post-anthesis K translocation compared to other hybrids.

### Nutrient uptake in the shoot (above-ground) and efficiencies

Compared to late sown, the timely sown crop had a greater uptake of shoot N by 15.6% (2020) and 29.3% (2021) (Table 4). The hybrid AH-4271 was most effective when it came to absorbing N in the shoot. PMH-1 and AH-8181 recorded the highest values for shoot P uptake. Further, timely sown hybrids had 21.8% and 32.2% greater shoot K uptake than the late sown hybrids. In both the years, hybrids PMH-1, AH-4271 and PJHM-1 showed a greater shoot K uptake. Timely sown maize had 15.8–29.5% greater N uptake efficiency (NupE) than the late sown maize. In both the years, AH-4271 was the most efficient hybrid in absorbing N. On the other hand, the timely sown crop had a greater P uptake efficiency (PupE) than the late sown crops. The P uptake efficiency (PupE) in hybrids was greater for PJHM-1 in 2020, but for PMH-1 and AH-8181 in 2021. The K uptake efficiency (KupE) under timely sown was 21.7–32% greater than the late sown crop. PMH-1 and PJHM-1 gave the maximum K uptake efficiency in both years.

**Table 4:** Above ground nutrient uptake and nutrient uptake efficiencies of maize genotypes under timely and late sown conditions.

Treatment	AGN (kg ha^−1^)		AGP (kg ha^−1^)		AGK (kg ha^−1^)		NupE (kg Kg^−1^)		PupE (kg Kg^−1^)		KupE (kg Kg^−1^)	
	2020	2021	2020	2021	2020	2021	2020	2021	2020	2021	2020	2021
Sowing time (S)												
Timely	215.3a	229.7a	18.8a	20.8a	119.8a	133.4a	0.57a	0.61a	0.34a	0.37a	0.23a	0.25a
Late	181.7b	162.3b	16.2a	17.9a	93.6b	90.5b	0.48b	0.43b	0.29a	0.32a	0.18b	0.17b
Genotypes (G)												
PMH 1	213.2a	177.9b	18.8a	16.8b	114.8a	107.7b	0.56a	0.47b	0.34a	0.30b	0.22a	0.21b
PJHM 1	172.9c	201.9a	16.2b	21.6a	98.1c	119.9a	0.46c	0.53a	0.29b	0.39a	0.19c	0.23a
AH 4158	204.5ab	206.4a	17.5ab	17.1b	102.8c	107.6b	0.54ab	0.54a	0.30ab	0.31b	0.20c	0.21b
AH 4271	219.6a	208.5a	17.8ab	20.0ab	112.7ab	110.7b	0.58a	0.55a	0.32ab	0.36ab	0.21ab	0.21b
AH 8181	182.2bc	185.5b	17.1ab	21.1a	105.1bc	113.7ab	0.48bc	0.49b	0.31ab	0.38a	0.20bc	0.22ab
S × G	ns	ns	**	*	**	*	ns	ns	**	*	**	**

AGN: Total above ground nitrogen uptake, AGP: Total above ground phosphorus uptake, AGK: Total above ground potassium uptake, NupE: Nitrogen uptake efficiency, PupE: Phosphorus uptake efficiency, KupE: Potassium uptake efficiency. Within, sowing time and genotypes, different letters indicate the significant difference (p ≤ 0.05); The S × G implies the interaction between sowing time and genotypes; **Significant at (*p* ≤ 0.01); *Significant at *p* ≤ 0.05; ns: not significant.

### Nutrient (N, P and K) use efficiencies

**Figure 6:**
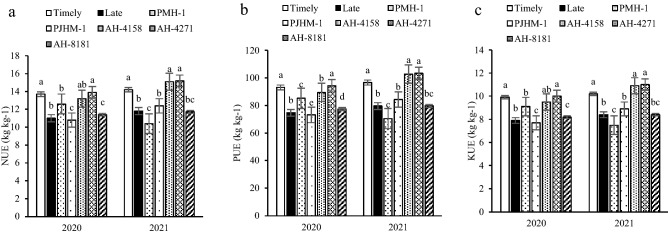
NUE- nitrogen use efficiency (**a**), PUE- phosphorus use efficiency (**b**), KUE- potassium use (**c**). Within years, sowing time, and genotypes, different letters indicate significant difference (*p* ≤ 0.05).

The N use efficiency (NUE, kg kg^−1^) under timely sown conditions was 16.9–19.9% greater than the late sown conditions (Fig. 6a). Also, AH-4158 and AH-4271 recorded the greater NUE. Timely sowing had 17.6–19.8% greater P use efficiency (PUE kg kg^−1^). Hybrid AH-4271 had the highest PUE (Fig. 6b). Under timely sowing, KUE (kg kg^−1^) increased by 17.6–20.2% than the late sown conditions. Again, AH-4271 had the greater KUE and being similar to the AH-4158 (Fig. 6c).

### Stress tolerance indices

In terms of grain and stover yields, maize hybrids exhibited a variable response to the varying stress tolerance indices (Tables 5, 6). The maximum grain yield reduction of 25.4% was recorded with the hybrid AH-4271. The most stable hybrids for grain yield, however, were AH-4158 and AH-8181. Furthermore, these hybrids also exhibited the least grain stress susceptibility index (SSI). The advanced hybrids AH-4271 (5.52 t ha^−1^) and AH-4158 (5.36 t ha^−1^) had the highest grain mean productivity (MP) and grain geometric mean productivity (GMP), respectively. In addition, the highest stover yield reduction was recorded with the AH-8181 (25.9%) due to the late planting. In terms of stover yield stability index (YSI), AH-4158 and AH-8181 were comparatively more stable with varying sowing times. In contrast, the hybrids with the highest stress susceptibility were PMH-1 and AH-8181. Nevertheless, the MP (7.45 t ha^−1^) and GMP (7.37 t ha^−1^) were highest with the hybrid PMH-1.

**Table 5:** Grain stress tolerance indices of maize genotypes under timely and late sown conditions.

Genotypes	*Average yield (Mg ha^−1^)					(Mg ha^−1^)	
	Timely	Late	%YR	YSI	SSI	MP	GMP
PMH 1	4.80 ± 0.38	3.90 ± 0.26	18.79 ± 1.01	0.81 ± 0.01	1.01 ± 0.05	4.35 ± 0.32	4.33 ± 0.31
PJHM 1	4.81 ± 0.23	3.97 ± 0.14	17.25 ± 2.88	0.83 ± 0.07	0.93 ± 0.36	4.39 ± 0.06	4.37 ± 0.05
AH 4158	5.80 ± 0.22	4.93 ± 0.38	15.15 ± 1.17	0.85 ± 0.04	0.81 ± 0.21	5.36 ± 0.30	5.35 ± 0.30
AH 4271	6.31 ± 0.31	4.73 ± 0.25	25.07 ± 0.94	0.75 ± 0.01	1.35 ± 0.05	5.52 ± 0.28	5.46 ± 0.28
AH 8181	4.75 ± 0.11	4.02 ± 0.30	15.24 ± 2.30	0.85 ± 0.08	0.82 ± 0.41	4.39 ± 0.12	4.37 ± 0.14

*Mean yield of 2 years; YR: Percentage yield reduction, YSI: Yield stability index, SSI- Stress susceptibility index, MP: Mean productivity, GMP: Geometric mean productivity.

**Table 6:** Stover stress tolerance indices of maize genotypes under timely and late sown conditions.

Genotypes	Average yield (Mg ha^−1^)*					(Mg ha^−1^)	
	Timely	Late	%YR	YSI	SSI	MP	GMP
PMH 1	8.48 ± 0.03	6.42 ± 0.57	24.25 ± 1.96	0.76 ± 0.07	1.40 ± 0.40	7.45 ± 0.27	7.37 ± 0.32
PJHM 1	7.90 ± 0.14	6.50 ± 0.07	17.73 ± 1.77	0.82 ± 0.01	1.03 ± 0.04	7.20 ± 0.10	7.17 ± 0.10
AH 4158	7.45 ± 0.22	6.20 ± 0.13	16.70 ± 1.17	0.83 ± 0.04	0.97 ± 0.24	6.83 ± 0.04	6.80 ± 0.03
AH 4271	7.71 ± 0.39	6.73 ± 0.36	12.72 ± 1.26	0.87 ± 0.02	0.74 ± 0.02	7.22 ± 0.37	7.20 ± 0.37
AH 8181	8.33 ± 0.09	6.17 ± 0.21	25.91 ± 1.40	0.74 ± 0.03	1.50 ± 0.20	7.25 ± 0.06	7.17 ± 0.08

*Mean yield of 2 years; %YR: Percentage yield reduction, YSI: Yield stability index, SSI: Stress susceptibility index, MP: Mean productivity, GMP: Geometric mean productivity.

### Root system traits

**Figure 7:**
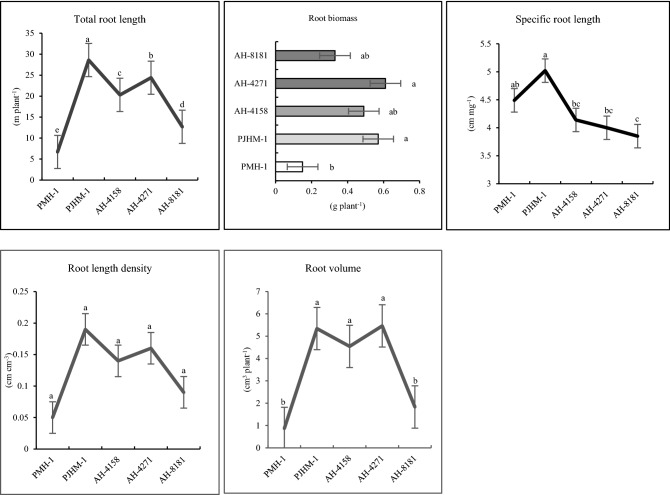
Root morphological traits of five maize hybrids grown in PVC tubes under field condition at V_5_ stage (20 DAS). Means followed by different letters on the individual bars/lines of a figure indicate significant difference (*p* ≤ 0.05).

**Figure 8:**
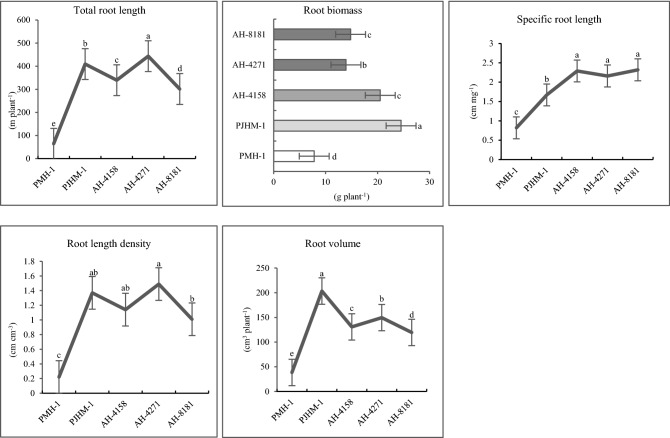
Root morphological traits of five maize hybrids grown in PVC tubes under field condition at 50% flowering (53 DAS). Means followed by different letters on the individual bars/lines of a figure indicate significant difference (*p* ≤ 0.05).

The maize hybrids had a significant variation in the root system traits, such as, root length (RL), root biomass (RB), root length density (RLD), and specific-root length (SRL). PJHM-1 had the highest root length, RLD, and SRL at the 20 DAS, while the maximum root biomass and volume was recorded with the advanced hybrid AH-4271 (Fig. 7). At 50% flowering (53 DAS), AH-4271 had the maximum RL, RLD, and SRL (Fig. 8), however, PJHM-1 had greater RB compared to other hybrids.

## Discussion

Timely sowing of maize hybrids enhanced the yields through better crop stand, improved yield attributes coupled with higher post-anthesis accumulation of dry matter and nutrient uptake. The contribution of post-anthesis dry matter accumulation to grain yield under both the sowing dates was much higher than the contribution from dry matter translocation. Indeed, the contribution of dry matter translocation to grain yield under the late sown was more than the timely sown, thereby indicates the importance of stored assimilates before anthesis under stress environment. While, for nutrients (N and K), the contribution to grain yield from translocation was more compared to the uptake, but the reverse hold true for P. Dry matter translocation and accumulation, nutrients translocation and uptake, and their contribution to grain yields varied significantly among the hybrids. Further, hybrids with higher geometric mean productivity and tolerance index are more productive under the varying sowing dates. On an average, timely sowing had a 17.9% and 18.9% yield advantage for grain and stover yields, respectively (Table 1), which could be attributed to the better photosynthates partitioning within the plant, as the crop has an extended period of photosynthesis^4^, with more favourable weather conditions during the growth and development for a particular region^6^. In addition, timely sowing also enhances the synchronization of maximum green leaf area index and the peak solar radiation^31^, thereby, improved the intercepted photosynthesis rate and hence the crop development^32^, resulting in greater yield. Additionally, the higher yield of hybrids in 2021 could be attributed to higher rainfall and its better distribution, particularly during the reproductive stage (Suppl. Figure 1).

The delayed sowing had the negative impacts on yields, by reducing the kernel number and their weight^33^. Comparable findings for the reduced kernel number and weight have also been reported by^5^, wherein late sowing would not be able to establish a proper root system under stress conditions. Hence, the uptake and partitioning of water and nutrients under the late sowing couldn’t meet the crop requirement for proper growth and development during the reproductive stage, which might have led to the under developed kernel. Subsequently, delayed sowing could also reduce the number, size and activity of growing grains coupled with the decreased supply of assimilate to grains during the period of grain filling, hence the grain yield^31^. There was a strong correlation between kernel weight with temperature and solar radiation^34^, and kernel weight with the grain yield^31^. Further, the main cause of the reduction in grain yield under late sowing was the reduction in grain number^35^.

Post-anthesis dry matter accumulation under timely sown accounted for 62.5% (av. of 2 yrs.) of the grain yield, wherein it was 55.8% greater than the late sown crop. While, it ranged from 55.6 to 67.7% in 2020 and 48.1 to − 64.1% in 2021 among the hybrids (Fig. 1e,f), if respiratory losses for maintenance and remobilization of pre-anthesis accumulated assimilate are not taken into consideration^27^. The assimilates for grain formation don’t come entirely from the current assimilation which are directly transferred to the kernels, but also from the remobilization of temporarily stored assimilates in different vegetative plant parts^12^. Accumulated dry matter after anthesis is the major source for grain filling^13^, and in this study, the timely sown accumulated 17.2% higher dry matter and contributed 10.5% greater to the grain yield (Fig. 1c,d) over the late sown crop. This could in fact be associated with the congenial environment for growth and development^36^, particularly during the grain filling period. In addition, hybrids with greater post-anthesis dry matter accumulation had the positive effects on grain yield, though PMH-1 had relatively lower grain yield which could be ascribed to the lower number of cobs per unit area (Table 2).

The N, P, and K accumulated in vegetative organs of the crop before anthesis, remobilized for grain filling. However, unlike the dry matter, the N, P, and K uptake post-anthesis could not meet the requirement for grain development^27^. Indeed, this study outlined that, the larger amount of N and K uptake occurred before anthesis, while the reverse is true for P (Figs. 2a,b, 4a,b) irrespective of the sowing dates and hybrids. The N and P are incorporated into the leaves and along with K it takes part in photosynthesis, hence recycling and remobilization of the stored N, P, and K in the vegetative tissues pre-anthesis would affect the photosynthesis processes^27,28^. It has been reported in maize and wheat that a greater amount of N and P accumulated during pre-anthesis is remobilized and recycled under the N and P deficiency^37,38^.

In the present study, timely sown had higher post-anthesis uptake of N, P, and K (Figs. 2b, 3b, 4b) over the late sown crop, which could be the result of better growth and development of both above and the below-ground due to congenial environment, particularly during the grain filling period. Similar results of greater uptake of N, P, and K during the post-anthesis was reported by^39^. Further, the greater uptake of N, P, and K during the post-anthesis implies that it had the priority to be used in grain formation as can be visualized with the greater yields (Table 1). The greater post-anthesis uptake of N, P, and K under the timely sown crop is used to prolong the stay-green period of leaves, consequently, promotes more grain formation resulting in better yields^22,23^, but the grain % N was lower compared to the late sown crop (Table 3). Similar results of higher post-anthesis nutrient uptake with lower grain % N have also been reported by^20,21,27^.

The improvement in yields of maize hybrids under variable environmental conditions is desirable, hence in the present study various stress indices were employed to find out the best performing hybrid under the timely and late sown conditions. The higher rate of mean productivity (MP) and geometric mean productivity (GMP) coupled with a lesser stress susceptibility index (SSI) indicated that the genotypes had greater stress tolerance with the enhanced yield potential^15^. The hybrid AH-4158 had the least yield reduction and SSI with greater YSI (Table 5), thereby, it had greater tolerance with the time of sowing. Subsequently, AH-4271 outperformed for grain MP and GMP though it was more susceptible to late sowing. This hybrid AH-4271 would be well suited under the timely sown with higher productivity potential. In addition, this hybrid had greater stability for the stover yield with different sowing dates (Table 6).

A positive correlation was reported between SSI and grain yield of wheat genotypes to identify the best performing varieties under stress conditions^40^. Further, classifying the genotypes based on MP and GMP were similar, and had positive relations with the grain yield under normal and stress conditions^15,41^. The MP, which is the average productivity of yield under stress and normal conditions^42^, and its greater value denotes a better performance of hybrids under the stress conditions, hence a good criterion for selecting a hybrid tolerant to stress. The studies of drought stress on maize hybrids yield reported that under normal and mild stress conditions, the GMP, MP, and stress tolerance index (STI) were important indices for identifying the best performing hybrids under the variable environments^43^. Further, a positive correlation of grain yield with MP and GMP under severe stress and normal conditions were observed, thereby helps in determining the drought-tolerant hybrids^44^.

In our experiment, a hybrid with greater MP, GMP, and YSI could be used for identifying the hybrids adaptability to different sowing dates (timely and late). In terms of grain yield, the AH-4271 and AH-4158 had greater MP and GMP under the variable sowing dates, indicating their superiority. The adoption of N efficient hybrids is a vital management strategy for enhancing the N use efficiency (NUE)^45^. The NUE is grain yield per unit available-N both from soil and through applied fertilizer^16^. In cereals, the NUE has been reported to be about 40% of the applied fertilizers^46^. In our study, the NUE ranged between 10–15 kg kg^−1^ considering the contribution from 0.0–0.30 m soil profile in addition to the applied N fertilizer (Fig. 6a).

The greater NUE of AH-4271 and AH-4158 under the timely sown conditions could be attributed to the enhanced uptakes by their root coupled with the better assimilation and remobilization in the shoot^16,47^. Studies on wheat showed that NUE could be improved through optimization of the root system^48,49^. Furthermore, significantly greater P and K use efficiencies under timely sown (Fig. 6b,c) might be the result of better growth and development with higher grain yield due to the congenial crop environment. Subsequently, differential growth and development habits of the hybrids in changing environments lead to variation in P and K use efficiencies. The greater P and K efficiencies with the AH-4271 and AH-4158 (Fig. 6b,c), showed their responsiveness and superiority through better adaptation under the varied ecologies. The importance of growing nutrients use efficient genotypes has been emphasized, as it would reduce the excessive fertilizers input without yield penalty^16,49^.

In addition, the rooting traits varied among the hybrids (Figs. 7, 8), where in the hybrid with better root length and biomass coupled with greater root length density produced better yields (Table 1). The genotypes with better root proliferation at the early stage might have led to the better crop establishment and used the available resources more efficiently and partitioned proportionately to different plant parts, resulting in the greater output. Indeed, the greater number of cobs per unit area was recorded with those hybrids having better root morphological traits, thereby indirectly implies more crop stand on a unit area. Genotype with a greater root growth and proliferation proportionately partitioned the captured resources and thus enhanced the yields^29,30^. Similarly, studies on wheat showed that root biomass was positively correlated to the number of grains spike^−1^ and yields^50^, so was the case in the present study, i.e., a higher number of grains cob^−1^ and grain yields related.

## Conclusions

Timely sowing, a resource-saving practice plays a vital role in enhancing the yield potential of maize genotypes. Compared with late sown, timely sown yielded 16–19% more grain and produced 5–10% more cobs per hectare. During the grain filling period, post-anthesis dry matter accumulation is crucial, and it was significantly higher with the timely sowing compared to the late sowing. We observed maximum nutrient use efficiency, nutrient uptake, and nutrient contribution to grain yield under the timely sowing conditions. Advanced hybrids, AH-4271 and AH-4158, performed better in various parameters, viz., grain yield, nutrient uptake, and cobs per ha. Hybrid AH-4158 showed higher yield stability, a lower stress susceptibility index, and a lower percentage of grain yield reduction, indicating the enhanced capacity for flexibility in a variety of crop-growing conditions, together with hybrid AH-4271. Indeed, better root morphology correlates with the greater nutrient use efficiency, dry matter accumulation, and nutrient remobilization to produce higher yields. Therefore, assessing hybrids based on stress indices, nutrient remobilization, and grain yield could lead to identifying the best hybrids under the variable crop conditions.

## Materials and methods

### Experimental site and weather conditions

A Fixed-site field experiment was conducted for 2 years at the ICAR-Indian Agricultural Research Institute, New Delhi, India, planting maize genotypes under the timely and late sown conditions during the rainy seasons of 2020 and 2021. The region falls under the Trans Indo-Gangetic plains with 28°38′ N latitude, 77°10′ E longitude, and 229 m amsl. The climate is semi-arid with hot summers and rainy in monsoon (July–September), with scattered rains in winter. There is an annual mean rainfall of 650 mm, and the mean maximum and minimum temperatures range from 20–40 °C to 4–28 °C, respectively. The 2 years weather observations (2020–21) recorded by the automated observatory in the adjacent experimental site are summarized in suppl. Figure 9. Before preparatory tillage, the soil samples were collected randomly from 0.0–0.15 m to 0.15–0.30 m undisturbed soil depth. the samples were air-dried, ground, sieved through a 0.2 mm sieve, and stored in air-tight polyethene bags for further analysis of soil chemical properties, viz. soil pH (1:2.5, soil: water^51^), KMnO_4_-oxidizable N^52^, NaHCO_3_ extractable P^53^, NH_4_OAc exchangeable K^54^, and soil organic carbon (S_OC_)^55^ (Table 7).

**Table 7:** Initial soil chemical properties of the experimental site.

Soil chemical properties	Content	Analysis methods
Soil pH (1:2.5, soil: water)	8.1	Piper (1950)
Soil organic carbon (SOC)		
a) 0.0–0.15 m	4.66 (g kg^−1^)	Walkley and Black (1934)
b) 0.15–0.30 m	3.39 (g kg^−1^)	
KMnO_4_-oxidizable N		Subbiah and Asija (1956)
a) 0.0–0.15 m	150 (kg ha^−1^)	
b) 0.15–0.30 m	78.4 (kg ha^−1^)	
0.5 N NaHCO_3_ extractable P		Olsen et al. (1954)
a) 0.0–0.15 m	15.4 (kg ha^−1^)	
b) 0.15–0.30 m	14.2 (kg ha^−1^)	
0.1 N NH_4_OAc exchangeable K		Hanway and Heidel (1952)
a) 0.0–0.15 m	288.1 (kg ha^−1^)	
b) 0.15–0.30 m	188.9 (kg ha^−1^)	

### Cultural operations, experimental design, and crop management

Pre-sowing irrigation was applied before preparatory tillage operations. The field was deep ploughed twice using a disc harrow (0.00–0.20 m depth) followed by planking with a rotavator/ cultivator twice and finally levelled. The experiment was laid out in a split-plot design with three replicates. Two sowing times (timely and late sown) were allocated to the main plots and five recently released maize hybrids (PMH-1, PJHM-1, AH-4158, AH-4271, and AH-8181) to sub-plots, with a sub-plot size of 20 m^2^ (4 m × 5 m). In 2020, the crop was sown on 6th July (timely) and 27th July (late), while in 2021 it was sown on 3rd July (timely) and 24th July (late), respectively. The seeds were dibbled manually at a spacing of 0.75 m (row–row) × 0.20 m (plant–plant) in both the seasons. Earthing-up was done at the knee-high stage for better crop growth, prevent lodging and uniform distribution of irrigation water. Based on the critical growth stages and the rainfall received during the crop seasons, irrigation water was applied to a depth of 0.05 m. The recommended fertilizer application rate for maize was 150:26.2:49.59 kg NPK ha^−1^. Nitrogen (N) was applied as urea (46% N), phosphorus (P) and potassium (K) through di-ammonium phosphate (46% P_2_O_5_), and muriate of potash (60% K_2_O), respectively. At sowing, full doses of P, K, and 50% N were applied uniformly in all the plots, while the remaining 50% N was top-dressed in two equal splits at knee-high and tasseling stages. Weeds were controlled through broad-spectrum pre-emergence herbicide atrazine (50% WP, at 750 g a.i. ha^−1^) applied a day after sowing, followed by one hand weeding at 35–40 days after sowing (DAS). For insect-pests management, particularly fall armyworm (*Spodoptera frugiperda*), a systemic insecticide emamectin benzoate (50% SG, at 200 g ha^−1^) was first sprayed at 20 DAS, followed by need-based at 15–20 d intervals on their appearance. The insect-pests and disease management were carried out uniformly in all the plots based on the recommended practices.

### Plant sampling and their laboratory analysis

Plant samples were collected at two stages of the crop growth (tasseling and maturity) at two different dates in each season. In each plot, three plants were randomly cut at the base and separated into leaf, stalks (stem + leaf sheaths + tassel), and leaf, stalks (stem + leaf sheaths + tassel + husk), cob and grain at maturity. The samples were placed in a perforated brown paper bag, air-dried for 48 h, and then oven-dried at 65 ± 2 °C to a constant weight. The dry matter (DM) values were used to determine the translocation, accumulation, and efficiency as per the equations described by (1)^11,21,56^. A Macro Wiley-mill having a 40-mesh sieve was used for grinding the plant samples, and appropriate amounts (0.5 g) of the ground samples were used to determine the total N concentration employing the modified Kjeldahl digestion process, total P by colored Vanado-molybdo-phosphoric acid procedure, and total K by flame photometer method^57^. The nutrient translocation, uptake, and efficiency were computed in accordance with the Eq. (2)^21,58^. The soil available nutrients in this study were determined to the depths of 0.00–0.30 m. Also, the above-ground nutrient uptake at maturity, uptake efficiencies were computed by using Eq. (3)^59,60^. The above-ground nutrient uptake was worked out by multiplying the nutrient concentration in stalks and grains with the biomass yields. To estimate the uptake and use efficiencies, the soil nutrients available to a depth of 0.30 m (0.00–0.15 m, 0.15–0.30 m) were used.

**Table:** 

DM translocation, accumulation and their efficiencies	(1)
i. Pre DMT (DM translocation before anthesis; g plant^−1^) = DM (total above-ground) at anthesis − DM (vegetative parts) at maturity	
ii. Pre DMTe (DM translocation efficiency before anthesis; %) = (Pre-DMT/DM at anthesis) × 100	
iii. Post DMA (DM accumulation after anthesis, g plant^−1^) = DM (total aboveground) at maturity − DM at anthesis	
iv. Post DMAe (DM accumulation efficiency after anthesis; %) = (Post-DMA/DM at maturity) × 100	
v. Pre DMTg (Contribution of DM translocation before anthesis to grain yield; %) = (Pre DMT/grain yield) × 100	
vi. Post DMAg (Contribution of DM accumulation after anthesis to grain yield; %) = (Post DMA/grain yield) × 100

**Table:** 

Nutrients translocation, uptake and their efficiencies	(2)
i. Nutrients translocation before anthesis (Pre NT, Pre PT, Pre KT; mg plant^−1^) = Total above-ground nutrient (N/P/K) at anthesis − total nutrient (vegetative, N/P/K) at maturity	
ii. Nutrients translocation efficiency before anthesis (Pre NTe, Pre PTe, Pre KTe; %) = [(Pre NT, Pre PT, Pre KT)/Total above-ground nutrient (N/P/K) at anthesis] × 100	
iii. Nutrients uptake after anthesis (Post Nup, Post Pup, Post Kup; mg plant^−1^) = Total above-ground nutrient (N/P/K) at maturity − nutrient (vegetative, N/P/K) at anthesis	
iv. Ratio of nutrients uptake after anthesis to total nutrients accumulation (Post NR, Post PR, Post KR; %) = [(Post Nup, Post Pup, Post Kup)/(total nutrient (N/P/K) accumulation)] × 100	
v. Contribution of nutrients translocation before anthesis to grain yield (Pre NTg, Pre PTg, Pre KTg; %) = [(Pre NT, Pre PT, Pre KT)/(grain nutrient (N/P/K) at maturity)] × 100	
vi. Contribution of nutrients uptake after anthesis to grain yield (Post NupG, Post PupG, Post KupG; %) = [(Post Nup, Post Pup, Post Kup)/(grain nutrient (N/P/K) at maturity)] × 100	

Pre NT, Pre NTe, Post Nup, Post NR, Pre NTg, Post NupG- Nitrogen; Pre PT, Pre PTe, Post Pup, Post PR Pre PTg, Post PupG- Phosphorus; Pre KT, Pre KTe, Post Kup, Post KR, Pre KTg, Post KupG- Potassium.

**Table:** 

Nutrient uptake and use efficiencies	(3)
i. Above-ground nutrients uptake (AGN/ AGP/ AGK; kg ha^−1^) = Total above-ground nutrients uptake at maturity	
ii. Uptake efficiency (NupE/ PupE/ KupE; kg kg^−1^) = [AGN, AGP, AGK/available N, P, K (soil + fertilizer)]	
iii. Use efficiency (NUE/ PUE/KUE; kg kg^−1^) = [Grain yield ha^−1^/ available N, P, K (soil + fertilizer)]	

AGN, NupE, NUE—Nitrogen; AGP, PupE, PupE—Phosphorus; AGK, KupE, KUE—Potassium.

### Stress tolerance indices

The maize genotypes were subjected to mathematical relationships on the basis of stover and grain yields for identifying the best performing genotype under normal and the delayed sowing conditions. The following formulae were used to find out the efficient genotypes^15,16^.

i. Percentage reduction of yield (YR; %) = (Y_T_ − Y_L_)/(Y_T_ × 100)
ii. Mean productivity (MP) = (Y_T_ + T_L_)/2
iii. Stress susceptible index (SSI) = (1 − Y_T_/Y_L_)/SI. Where Stress intensity (SI) was calculated as, SI = 1 − (X_T_/X_L_)
iv. Geometric mean productivity (GMP) = √(Y_T_ × Y_L_)
v. Yield Stability Index (YSI) = Y_T_/Y_L_

Where, Y_T_ and Y_L_ are the yields of genotypes under timely and late sown, respectively. The X_T_ and X_L_ denote mean yield of all genotypes under timely and late sown conditions, respectively.

### Yield attributes and yields

The physiological growth stages were marked when 50% of the plants attained their particular stage, such as, tasseling, silking, and physiological maturity. In the first season, maize was harvested on 18th and 27th October 2020, while in the second season on 16th and 27th October 2021, respectively for the timely and late sown conditions. The crop was harvested from the middle three rows (4 m × 2 m, 8 m^2^) leaving two border rows on each side. First, the cobs were hand-picked, then the stover was cut from the ground surface. The harvested produce was sun-dried for 25–30 d to bring down the grain moisture from 20–22% to 14–15% for threshing and the yield measurement for grain and stover were done separately. Further, the yield attributes, such as, number of rows cob^−1^, number of grains cob^−1^, cob weight (g), grains weight cob^−1^ (g), and 100-grains weight (TW, g) were determined from the five randomly picked cobs.

### Root system traits

**Figure 9:**
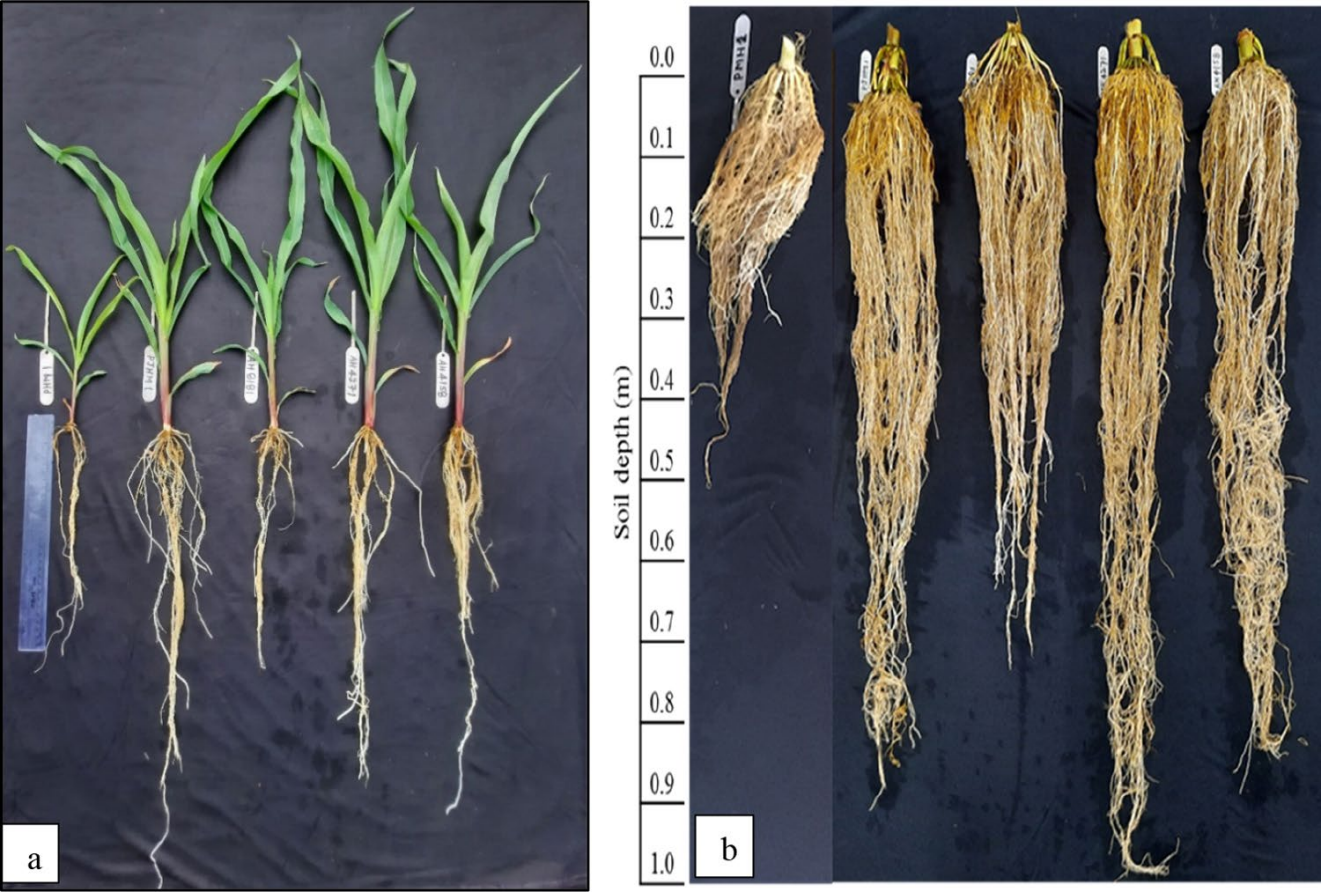
Destructively measured root growth pattern of the maize genotypes (PMH-1, PJHM-1, AH-8181, AH-4271, AH-4158) grown in PVC tubes under the field conditions at V5 stage (20 days after sowing, DAS) (**a**) and at 53 DAS (**b**).

The plants were grown in 25 mm PVC tube, 0.195 m diameter, 0.5 m and 1.0 m deep, transparent cylindrical polyethylene sheet placed on the inner PVC tube, filled with soil to a depth of 1.0 m. The PVC tubes were placed vertically on the steel stands in an open field condition. The soil (0.0–0.15 m depth) for the above purpose was collected from the experimental field of the current study. The 5 mm sieve was used to sieve out the debris and other undesirable materials before being packed into the PVC tubes. The soil in the tubes was manually watered carefully to saturation level so as to avoid the excess drainage (Pooniya et al.^30^). The soil in the tubes was fertilized at sowing to the depth of 0.01 m with an equivalent to the recommended fertilizer rate for maize hybrids (150:26.2:49.59 kg NPK ha^−1^), in the form of urea, diammonium phosphate, and muriate of potash. The plants were hand watered based on the critical growth stages as per the requirement. Three seeds each of the five recent/advanced hybrids were manually dribbled to a depth of 0.05 m in each tube, during the first week of July 2019. Five days after germination, a single plant was maintained in each tube after carefully thinning out the remaining seedlings. The five hybrids were grown in a completely randomized block design with three replicates and harvested twice {V5 stage-20 DAS (Fig. 9a) and 50% flowering- 53 DAS (Fig. 9b)}. At first sampling (0.5 m depth tube), the shoots were first cut from the roots at the crown, while in the second (1.0 m depth tube), the shoots and stilt roots above the soil were cut before washing. The polyethylene sheet was gently pulled from the tubes, cut open and dipped in the still water for an hour, repeatedly washed the soil on a 2 mm sieve to produce a clean root sample^29^. The recovered roots were placed in the plastic bags and stored at 5 °C until the scanning of its morphological traits. The scanned images of the roots were analyzed with WinRHIZO professional software (LA2400, Regent instrument, Quebec, Canada) for recording the root morphological traits (total root length and root volume, in the present study). The scanned roots were further dried in a hot air oven at 65 ± 5 °C until obtaining the constant weight for root dry biomass. The root length density was computed by dividing the total root length with the soil volume (0.0149 m^3^—1st sampling and 0.0298 m^3^—2nd sampling). While the specific root length was calculated as the total root length divided by the root biomass ^30^.

### Statistical analysis

With SAS 9.4, the data were statistically analyzed for split-plot designs through analysis of variance (ANOVA)^61^. In addition, Tukey’s honestly significant difference at 0.05 probability (*p *≤ 0.05) was used to compare the mean effects of the treatments i.e., sowing dates and genotypes.

Authors have confirmed that all the plant studies were carried out in accordance with relevant national, international or institutional guidelines.

## Supplementary Information

Supplementary Information.

## Data Availability

The data that support the findings of this study are available on request from the corresponding author. The data are not publicly available due to private and ethical restrictions.
