# Supplementary material for: Timely sown maize hybrids improve the post-anthesis dry matter accumulation, nutrient acquisition and crop productivity
Source: Sci Rep. 2023 Jan 30;13:1688. doi: 10.1038/s41598-023-28224-9 (PMC9886954; doi:10.1038/s41598-023-28224-9)
Supplement: Supplementary file 1 — Supplementary Information. [file 41598_2023_28224_MOESM1_ESM.docx]

a

**Supplementary fig. 1** Weekly mean temperature and relative humidity (a), precipitation and evaporation (b) during the cropping seasons of 2020 and 2021.
